# Systematic Phytochemical Screening of Different Organs of *Calotropis procera* and the Ovicidal Effect of Their Extracts to the Foodstuff Pest *Cadra cautella*

**DOI:** 10.3390/molecules26040905

**Published:** 2021-02-09

**Authors:** Ammar Bader, Ziad Omran, Ahmed I. Al-Asmari, Valentina Santoro, Nunziatina De Tommasi, Massimiliano D’Ambola, Fabrizio Dal Piaz, Barbara Conti, Stefano Bedini, Majed Halwani

**Affiliations:** 1 Department of Pharmacognosy, Faculty of Pharmacy, Umm Al-Qura University, Makkah 21955, Saudi Arabia; ambader@uqu.edu.sa (A.B.); zhomran@uqu.edu.sa (Z.O.); 2 King Abdullah International Medical Research Center, King Saud Bin Abdulaziz University for Health Sciences, Riyadh 11481, Saudi Arabia; 3 King Abdulaziz Hospital, Laboratory Department, Jeddah 6470, Saudi Arabia; 4 Dipartimento di Farmacia, Università di Salerno, 84084 Fisciano, Italy; vsantoro@unisa.it (V.S.); detommasi@unisa.it (N.D.T.); mdambola@unisa.it (M.D.); fdalpiaz@unisa.it (F.D.P.); 5 Department of Agriculture, Food and Environment, University of Pisa, Via del Borghetto 80, 56124 Pisa, Italy; stefano.bedini@unipi.it

**Keywords:** *Calotropis procera*, ovicidal activity, *C. cautella*, botanical insecticides, green insecticides

## Abstract

In developing countries, crop deterioration is mainly caused by inappropriate storage conditions that promote insect infestation. Synthetic pesticides are associated with serious adverse effects on humans and the environment. Thus, finding alternative “green” insecticides is a very pressing need. *Calotropis procera* (Aiton) Dryand (Apocynaceae) growing in Saudi Arabia was selected for this purpose. LC-MS/MS analysis was applied to investigate the metabolic composition of different *C. procera* extracts. Particularly, *C. procera* latex and leaves showed a high presence of cardenolides including calactin, uscharidin, 15β-hydroxy-calactin, 16β-hydroxy-calactin, and 12β-hydroxy-calactin. The ovicidal activity of the extracts from different plant organs (flowers, leaves, branches, roots), and of the latex, against *Cadra cautella* (Walker) (Lepidoptera, Pyralidae) was assessed. Extracts of *C. procera* roots displayed the most potent activity with 50% of *C. cautella* eggs not hatching at 10.000 ppm (1%).

## 1. Introduction

One third of the crops produced worldwide are estimated to be lost in the postharvest period. In developing countries, this loss is mainly caused by inappropriate storage conditions that promote insect infestation, and insect pests are responsible for losses as great as 40% in grain crops [1,2]. For this reason, chemical insecticides have been widely used to protect grains from insect attacks, but the serious adverse effects of synthetic pesticides on humans are a major health concern. Acute exposure to chemical insecticides can lead to respiratory tract issues, skin and eye irritation, allergy, extreme weakness, and seizures, while chronic exposure increases the incidence of Parkinson’s disease and cancer [3,4]. Consequently, in recent years, many synthetic insecticides—such as chlorine, some organophosphorus compounds, and some carbamate derivatives—have been banned in various countries [5].

Conversely, botanical insecticides containing active components derived from plant extracts are considered a safe and environmentally friendly option for integrated pest management (IPM) [6]. The rationale for using botanical insecticides for pest control is that plants are able to defend themselves against their insect enemies by producing bioactive substances [7,8], and these compounds can be extracted and utilized on crops. The main advantage of botanical insecticides is that they can control insect pests while minimizing the side effects on non-target organisms and maintaining an ecological balance. In addition, botanical compounds decompose more quickly and easily than their synthetic counterparts.

One source of natural insecticides is *C. procera* (Aiton), a plant belonging to the Apocynaceae family. It is native to northern and tropical Africa, western and southern Asia, Indochina, and the Middle East [9]. It is characterized by a wide range of pharmacological properties [10,11] and is used in traditional medicine for the treatment of various diseases ranging from ulcers to leprosy, as well as spleen and liver diseases [12]. Its extracts have anti-bacterial, anti-inflammatory, and analgesic effects [13]. Moreover, *C. procera* also shows larvicidal activity against disease-carrying mosquitoes, such as *Culex quinquefasciatus*, *Anopheles stephensi* Liston, *Culex tritaeniorhynchus* Giles, and *Culex gelidus* (Theobald) (Diptera: Culicidae) [14,15,16,17,18], as well as repellent and oviposition deterrent effects against *Anopheles arabiensis* and *Culex quinquefasciatus* [19]. *C. procera* latex and its flavonoids were also shown to negatively affect the feeding behavior of the lesser grain borer, *Rhyzopertha dominica* (Fabricius) (Coleoptera: Bostrichidae) [20]. Furthermore, *C. procera* has insecticidal effects against foodstuff insects that cause food deterioration, such as *Tribolium confusum* and *T. castanum* [21,22].

*C. procera* is a desert plant that is native to and available in large quantities in Saudi Arabia [23]. Therefore, the aim of this work was to study the phytochemical profile of extracts from the flowers, leaves, branches, roots, and latex of *C. procera*. Extracts prepared from the different plant parts were analyzed and tested for their ovicidal activity against the almond moth, *C. cautella* (Walker) (Lepidoptera: Pyralidae), a stored-product pest that infests grains and dried fruits. To the best of our knowledge, no previous work had investigated the effects of *C. procera* extracts on this pest.

Plants like *C. procera* produce many secondary metabolites with various physiological and biological activities that can include deterrent and antifeedant activity [24]. Many plants with antifeedant properties have been evaluated as crop protectors and a source of green pesticides. These botanical insecticides are now proposed as attractive alternatives to synthetic chemical insecticides for pest management [25,26,27,28]. Botanical insecticides notably do not impose any threats to the environment or human health, unlike their synthetic chemical counterparts. However, the use of plants as botanical insecticides requires an availability of those plants on an industrial scale. One important example of a green pesticide plant species is the neem tree, *Azadirachta indica* A. Juss., which is extracted on a large industrial scale in India and around the world [29]. *C. procera* is also a good candidate as a green insecticide based on its deterrent effect against several insects.

In the present study, we hypothesized that *C. procera* is a potential commercial source of biodegradable insecticide because of its extensive distribution and plentiful biomass in temperate and tropical regions. Mass production on an industrial scale is feasible and could satisfy the increased demand for greener insecticides. Therefore, the aim of this study was to extract natural insecticide products to control insect and pest populations without affecting the ecological balance [30,31,32,33]. Here, liquid chromatography-mass spectrometry (LC-MS) analysis of the chemical contents from different organs and latex of *C. procera* revealed the presence of 37 constituents belonging to the cardenolide and flavonoid classes. The evaluation of the ovicidal activity on *C. cautella* eggs revealed that extracts of *C. procera* roots had the most potent activity and prevented the hatching of 50% of *C. cautella* eggs when administered at 10,000 ppm.

## 2. Results and Discussion

### 2.1. Chemical Characterization of Extracts

**Figure 1:**
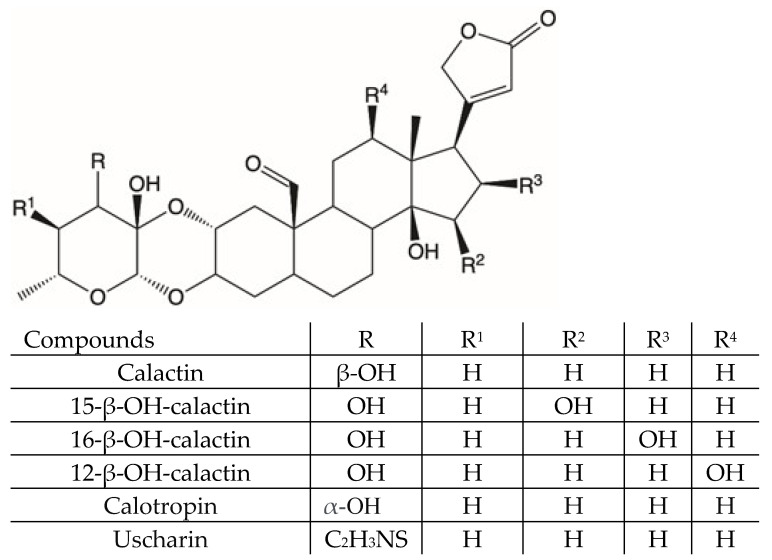
Cardenolides isolated from *Calotropis procera* leaves.

Previously published data suggest that the anti-microbial activity of *C. procera* extracts is related to their content in cardenolides [18]. In the present work, liquid chromatography coupled with high resolution mass spectrometry was used to study the metabolic composition of different organs of *C. procera*. By using liquid chromatography coupled with electrospray ionization to Orbitrap mass spectrometry technique (LC-ESI-Orbitrap-MS/MS), 37 compounds were identified or tentatively identified on the bases of their retention time, MS spectra, and MS fragmentation patterns. Thus, we carried out a preliminary nuclear magnetic resonance (NMR)-based investigation of the extracts to confirm the presence of such compounds (Figures S1–S5). The *n*-hexane extracts were found to contain mainly waxes and, therefore, they were not analyzed further. The methanol extracts showed the presence of cardenolides with aldehydic functions and flavonoid derivatives (Figures S1–S5). The methanol extract from the leaves, which was the most abundant, were subjected to phytochemical investigation to isolate pure compounds. The cardenolides that were isolated and characterized are reported in Figure 1.

### 2.2. LC-MS/MS Analysis

**Figure 2:**
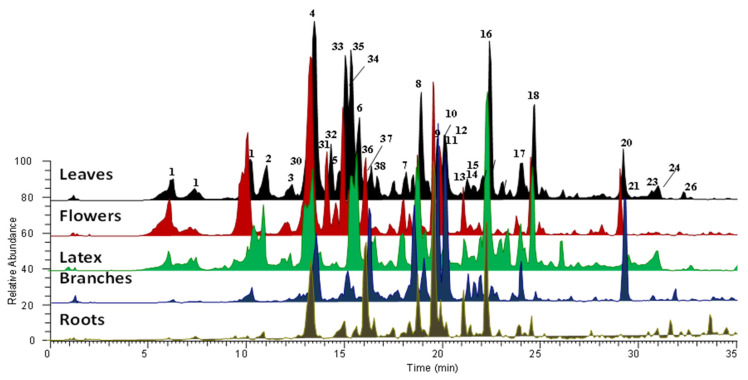
Extracted ion chromatograms, registered in positive ion mode, of methanolic extracts of different organs and latex of *C. procera.*

The methanolic extract of different plant organs (e.g., flowers, leaves, branches, roots), and the ethylacetate and butanolic extracts of latex, were then subjected to LC-MS/MS analysis in order to obtain a complete profile of the specialized metabolites and to compare their level in the extract. Resulting chromatograms, acquired in positive ion mode, are reported in Figure 2.

The identification of all compounds was based on the accurate mass value, mass fragmentation spectra, literature data comparisons, and, in some cases, by the use of pure compounds. Metabolites belonging to the flavonoid and cardenolide classes of chemicals were recognized (Table 1 and Figure 2). All of the organs and latex for *C. procera* from Saudi Arabia were found to be rich in cardenolides compounds, particularly calactin and its derivatives.

The flavonoid derivatives isorhamnetin, quercetin, and kaempferol were identified. Peaks 31, 33, 35, and 36 in the MS/MS spectra showed the same fragmentation ion at an *m/z* value of 317, which corresponded to isorhamnetin aglycone. Thus, based on the MS, MS/MS fragmentation, and literature data [42], these compounds were identified as isorhamnetin-hexoside-pentoside, isorhamnetin rutinoside, isorhamnetin robinoside, and two isorhamnetin hexoside isomers, respectively. Peak 30 was identified as rutin by a direct comparison to a reference compound. Finally, peaks 32, 34, and 37 corresponded to kaempferol derivatives, as their MS/MS spectra showed the same fragment ion at a *m/z* value of 287. These compounds were supposed to be kaempferol-rutinoside, kaempferol-robinoside, and kaempferol-glucoside; a subsequent injection of standard compounds led to a confirmation of these structures.

Compounds **1**–**29** were tentatively identified as cardenolides by comparing their HPLC elution order, HR-MS data, and HR-MS/MS data with previously reported data (Figure 2, Table 1) [34,35,36,37,38,39,40,41,42,43,44,45,46,47,48]. The cardenolide compounds exhibited very similar MS/MS spectra, i.e., several losses of H_2_O and CO, and for the glycoside compound, loss of the sugar unit [50]. Compounds **2**, **3**, **6**, **8**, and **10** showed a pseudo molecular ion at an *m/z* of 549.2622, which corresponded to the molecular formula of C_29_H_40_O_10_. Due to the MS/MS experiments, it was possible to assign different isobar compounds. Peaks 2, 3, and 6 exhibited the same fragmentation pathways. By comparison with reference samples, we were able to assign these peaks to isomers of hydroxy-calactin carrying the hydroxyl group in different positions (12, 15, and 16-hydroxycalactin). The fragment ion at *m*/*z* 405 in the MS/MS spectrum of the compound in peak 10, corresponding to a loss of the acidic residue, confirmed the presence of calactinic acid in the structure of this molecule. Finally, peak 8 was identified as calotoxin, and the MS/MS spectrum showed a loss of a hexose. Compounds **16** and **18** corresponded to calactin and calotropin, respectively. The fragment ion 387 detected in their MS/MS spectra was diagnostic of the presence of the aglycone calotropagenin (peak 4), as already observed in the MS/MS spectra of the previously described compounds (**8** and **10**). The presence of these analytes was confirmed by comparison with the isolated compounds. The same approach was used to identify the compounds having calotropagenin as aglycone, specifically compounds **1**, **14**, **17**, **19**, **21**–**24**, and **26**–**29**, which differed from each other only in the glycone unit [35]. Compounds **5** and **9** had the same aglycone, coroglaucigenin (peak 11). According to the MS/MS spectra, we could assign these two metabolites to hydroxy-coroglaucigenin and frugoside (coroglaucigenin-rhamnoside) [35]. Peaks 7, 12, and 15 corresponded to the uzarigenin (peak 20) derivatives uzarin, desglucouzarin, and *C. procera* saponin I, respectively. Finally, peaks 13 and 16, showed a similar MS/MS spectra, suggesting the presence of the aglycone afrogenin, and were assigned to afroside and labriformine, respectively.

### 2.3. Quantitative Analysis

In order to evaluate the amount of metabolites in the different plant parts, some of the most representative isolated compounds, belonging to cardenolide and flavonoid classes, were used to perform quantitative analysis in the extracts from organs and latex of *C. procera.* For this purpose, a fast and efficient LC-MS/MS analytical method was developed, optimized, and validated. Due to the use of the multiple-reaction monitoring (MRM) detection mode, it was possible to reach satisfactory lower limits of quantization for all compounds (see Material and Methods).

**Figure 3:**
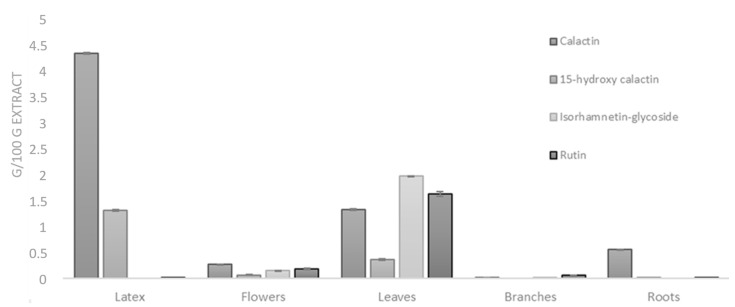
Amounts (g/100 g extract) of calactin, 15-hydroxy calactin, isorhamnetin glycoside, and rutin measured in different organs and latex of *C. procera* via multiple-reaction monitoring (MRM)-based LC-MS/MS analyses.

Quantitative analyses were carried out on different *C. procera* methanol extracts (latex, leaves, roots, branches, flowers). The mean values (±SD) calculated on the basis of the results obtained in at least three experiments showing similar results are reported (Figure 3). This analysis revealed that flavonoids were very abundant in the leaves (particularly isorhamnetin derivatives), while higher concentrations of cardenolide compounds were measured in the latex extract.

### 2.4. C. procera Extracts Ovicidal Activity

**Figure 4:**
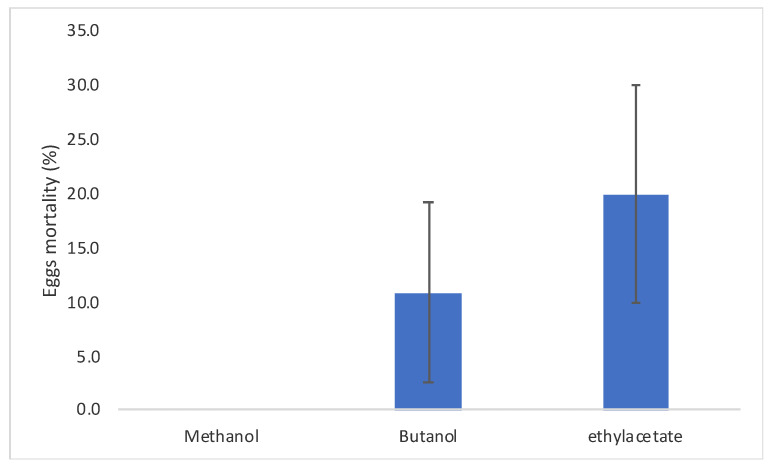
*Cadra cautella* eggs mortality when treated with the *C. procera* latex extracts. Bars represent standard error.

The ovicidal activity of *C. procera* latex extracts ranged from 0% to 20% for methanolic and ethyl acetate extracts, respectively (Figure 4), with no significant differences among the extractants (*F*_2,8_ = 1.75; *P* = 0.252). This data was probably linked, according to the LC-MS analyses, to the presence of some cardenolides present, mainly in the ethyl acetate extract of latex such as compounds **19**, **21**, **25,** and **27** (Table 1).

**Figure 5:**
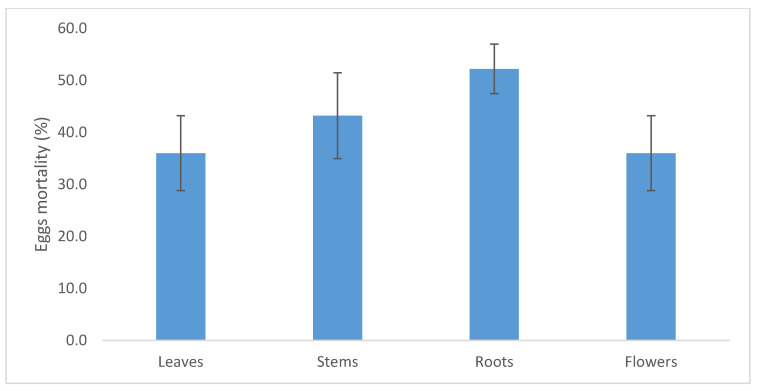
Mortality of eggs of *Cadra Cautella* treated with the *C. procera* methanolic extracts of leaves, branches, roots, and flowers. Bars represent standard error.

As for the different plant organ extracts, the methanolic extracts of *C. procera* roots showed an ovicidal activity ranging from 36% to 52% of the treated eggs for leaves and roots extracts, respectively (Figure 5), with no significant differences among the plant organ extracts (*F*_3,11_ = 1.17; *P* = 0.378).

In this research, *C. procera* root extracts showed clear ovicidal activity (50%). This finding is in line with the ovicidal activity of extracts from *Acorus calamus* (Araceae) (64%) roots. Further, it was higher than the Lamiaceae *Vitex negundo* (39%), *Adhatoda vasica* (15%) extracts, and *Dioscorea deltoidea* (Dioscoreaceae) (14%) extracts at a similar concentration (1.25%) against *Plutella xylostella* (Lepidoptera:Plutellidae) [51]. Similarly, *Plantago lanceolata* (Plantaginaceae) and *Momordica charantia* (Cucurbitaceae) methanolic extracts at 0.9% against *Leucoptera coffeella* (Lepidoptera: Lyonetiidae) eggs showed an ovicidal activity of 27% and 22%, respectively [52].

## 3. Materials and Methods

### 3.1. Plant Material

In 2017, *C. procera* plant material was collected in Makkah Al-Mukarramah between Arafat and Muzdalefah, Saudi Arabia (GPS coordinates 21°12′53.1′′ N, 40°17′05.3′′ E, altitude 286 m above sea level). Latex was collected by cutting the young and green stems. Leaves, branches, and flowers were collected from 20 plants. Roots were obtained from three middle sized plants. Plant organs were separately dried in shadows. The dried plant material was ground finely before extraction. All solvents used in the experiment were purchased from Sigma-Aldrich.

### 3.2. Extraction Methods

In this study, 50 g of each type of plant material—powdered leaves, flowers, stems, and roots—was extracted with 300 mL MeOH, followed by sonication for 30 min and centrifugation for 10 min at 2000 rpm. The supernatant was collected and evaporated under a vacuum. The latex was fractioned using a separatory funnel. Ethyl acetate was added and the mixture was vigorously shaken. After complete separation, the ethyl acetate layer was collected and filtered through filter paper to get rid of any latex traces. Then, the ethyl acetate extract evaporated under a vacuum using a rotary evaporator. The remaining latex was extracted with *n*-butanol by vigorous shaking. After complete separation of the two layers, the *n*-butanol fraction was evaporated under a vacuum using the rotary evaporator. Finally, methanol was added to the latex. After shaking, the methanolic layer was separated and evaporated as described above. The extract was separated on a silica gel column, the obtained fractions were further purified by reverse phase high performance liquid chromatography (RP-HPLC), and the isolated compounds were characterized by NMR experiments.

### 3.3. General Experimental Procedures

Optical rotations were measured by an Atago AP-300 digital polarimeter with a 1 dm microcell and sodium lamp (589 nm). NMR experiments were recorded on a Bruker Digital Receiver uniX 600 spectrometer at 300 K (Bruker BioSpin, Germany), revealing the spectra for methanol-d4 and CDCl_3_. HRESIMS data were obtained by using the positive ion modes on an Linear trap quadrupole LTQ Orbitrap XL mass spectrometer (Thermo Fisher Scientific). Quantitative data were acquired using an API6500 Q-Trap (ABSciex Foster City, CA, USA) apparatus.

### 3.4. Qualitative Analysis, LC-ESI-OrbitrapMS

The LC-MS method was applied to analyze specialized metabolites from different parts of *C. procera*. The adopted instrument configuration included an Accela (Thermo Fisher Scientific, Waltham, MA, USA) HPLC interfaced to a linear ion trap coupled with a high-resolution mass analyzer (LTQ-Orbitrap XL Thermo Fisher Scientific) through an Electrospray Ionization (EI) source. Separation was performed on a C18 column (Luna C18, Phenomenex, 100 × 2.0 mm, 2.5 µm) using a binary mobile phase composed of eluent A (ultrapure water–formic acid 0.1% *v*/*v*) and eluent B (ultrapure acetonitrile–formic acid 0.1% *v*/*v*). The separation conditions are 10% to 50% B within 40 min, followed by a second, faster gradient from 50% to 95% B within 10 min. The flow rate was 0.200 mL/min and the injection volume was 10.0 µL.

MS data were acquired in positive ion mode. At first, the full-mass and data-dependent scan mode was applied, and then tandem MS experiments were performed to identify the characteristic metabolites. The capillary temperature was set at 300 °C K, and the flow rate of sheath gas and auxiliary gas were set at 30.0 and 10.0 arbitrary units. The capillary voltage was 35.0 V, the source voltage was 3.5 kV, and the tube lens was 110 V. The mass resolution was set at 60,000.

### 3.5. Quantitative Analysis

The quantization of some isolated compounds, in particular calactin, 15-hydroxy calactin, rutin, and isorhamnetin-glycoside was carried out using an API6500 Q-Trap (ABSciex Foster City, CA, USA) coupled with a NexeraX2 UHPLC apparatus (Shimadzu, Kyoto, Japan). A positive multiple-reaction monitoring (MRM) system was selected for the LC-MS/MS analyses (Table 2). All the instrumental parameters were optimized by directly injecting solutions containing 10 mg/L of all of the pure compounds in methanol and water (50:50 *v*/*v*). All analyses of the MS data were performed in the positive ion mode. Samples of 5 μL were loaded onto a Luna Omega column (Phenomenex) (1.6 μm Polar C18 100 A, 50 mm × 2.1 mm), and compounds were separated using a linear gradient from 35% to 55% of acetonitrile (eluent B) in H_2_O containing 0.1% formic acid (eluent A) over 5 min. The flow rate was 0.4 mL min^−1^, and the injection volume was 5 μL for the standard compounds and latex extract samples. The total run time was 6 min. To perform accurate quantitative analyses, eight-point (0, 50, 150, 450, 1350, 4000, 12,000, and 24,000 ng/mL) calibration curves were built for the four purified compounds.

The linearity of the instrumental response as a function of sample concentration was confirmed for a wide range of concentrations (from 50 ng/mL to 24 μg/mL), and accuracy was fully satisfied (Table 2) for the investigated molecules.

### 3.6. Ovicidal Activity of C. procera Extracts on Cadra Cautella

The methanolic, butanolic, and ethyl acetate latex extracts were dissolved in methanol, *n*-butanol, and ethyl-acetate, respectively. Flower, leaf, root, and branch extracts were dissolved in DMSO 0.1% (aqueous solution). All solutions were tested at 10,000 ppm (1% concentration). Filter paper discs (5 cm diameter) treated with 100 μL of each extract solution, or solvent only for the controls, were placed inside a Petri dish (5 cm diameter). After the evaporation of the solvent, 50 eggs of *C. cautella* (0–24 h) were individually transferred onto the filter paper. Filter papers were kept wet with 50 μL of tap water. The test was replicated five times. The number of eggs unhatched was assessed after 24 h and calculated as a percentage of the total number of eggs. Experiments were conducted at 25 ± 1 °C, RH 65 ± 2%, and a photoperiod of 16:8 (L/D). *C. cautella* eggs mortalities were reported as a mean ± standard error (ES). Mortality was adjusted for the natural mortality of eggs [49] according to the following formula: Adjusted mortality (%) = 100 × (X−Y)/(100−Y), where X is the percentage mortality of the treated sample, and Y is the percentage mortality of the untreated control sample. Means were compared via one-way ANOVA with the extractant (latex extracts) or plant organ (plant extracts) as fixed factors. Equality of variances was checked before the analyses using the Levene’s test. Statistics were performed using SPSS 22.0 software (IBM SPSS Statistics, Armonk, North Castle, NY, USA).

## 4. Conclusions

In this study, we compared the phyto-constituents of different organs from an important medicinal plant widely distributed in the temperate regions, *C. procera*. In terms of large-scale extraction, this study could orient scientists when choosing organs rich in phyto-constituents. The title plant could be considered a source of biomolecules with potential anti-parasitic effects. Although the ovicidal activity is not as strong as that excreted by commercial synthetic molecules, our study contributes to the future development of safer and greener insecticides.

## Figures and Tables

**Table 1:** MS Data of Compounds **1**–**37** detected in leaves, latex, flowers, branches and roots extract of *C. procera*.

No	Formula	[M − H]^+^	MS/MS	Compound and CAS Number	Organs and Latex	Literature
**Cardenolides**						
**1**	C_23_H_32_O_7_	421.2209	403 [M+H-18]^+^ 385 [M+H-18-18]^+^ 367 [M+H-18-18-18]^+^ 339 [M+H-18-18-28-18]^+^	Hydroxycalotropagenin isomer ^b^ tentatively identified	Latex, flowers, leaves, branches, roots	[34]
**2**	C_29_H_40_O_10_	549.2622	531 [M+H-18]^+^ 513 [M+H-18-18]^+^ 485 [M+H-18-18-28]^+^ 467 [M+H-18-18-28-18]^+^ 385 [M+H-146-18]^+^ 367 [M+H-146-18-18]^+^ 339 [M+H-146-18-18-28]^+^	12-hydroxy-calactin ^a^ 2311818-49-0	Latex, flowers, leaves, branches, roots	standard
**3**	C_29_H_40_O_10_	549.2622	531 [M+H-18]^+^ 513 [M+H-18-18]^+^ 485 [M+H-18-18-28]^+^ 467 [M+H-18-18-28-18]^+^ 385 [M+H-146-18]^+^ 367 [M+H-146-18-18]^+^ 339 [M+H-146-18-18-28]^+^	15-hydroxy-calactin ^a^ 159406-83-4	Latex, flowers, leaves, branches, roots	standard
**4**	C_23_H3_2_O_6_	405.2254	387 [M+H-18]^+^ 369 [M+H-18-18]^+^ 341 [M+H-18-18-28]^+^ 323 [M+H-18-18-28-18]^+^	Calotropagenin^b^ 24211-64-1	Latex, flowers, leaves, branches, roots	[34,35,36,37,38,39]
**5**	C_23_H_34_O_6_	407. 2411	389 [M+H-18]^+^ 371 [M+H-18-18]^+^ 353 [M+H-18-18-18]^+^	Hydroxy coroglaucigenin isomer ^b^ tentatively identified	Latex, flowers, leaves, branches, roots	[40]
**6**	C_29_H_40_O_10_	549.2622	531 [M+H-18]^+^ 513 [M+H-18-18]^+^ 485 [M+H-18-18-28]^+^ 467 [M+H-18-18-28-18]^+^ 385 [M+H-146-18]^+^ 367 [M+H-146-18-18]^+^ 339 [M+H-146-18-18-28]^+^	16-hydroxy-calactin ^a^ 107110-13-4	Latex, flowers, leaves, branches, roots	standard
**7**	C_35_H_54_O_14_	699.3617	537 [M+H-162]^+^ 375 [M+H-162]^+^	Uzarin ^b^ 20231-81-6	Latex, flowers, leaves, branches, roots	[41]
**8**	C_29_H_40_O_10_	549.2622	531 [M+H-18]^+^ 513 [M+H-18-18]^+^ 485 [M+H-18-18-28]^+^ 467 [M+H-18-18-28-18]^+^ 387 [M+H-162]^+^ 369 [M+H-162-18]^+^ 351 [M+H-162-18-18]^+^ 323 [M+H-162-18-18-28]^+^	Calotoxin ^b^ 20304-49-8P	Latex, flowers, leaves, branches, roots	[34,35,38,39,40,42,43,44]
**9**	C_29_H_44_O_9_	537.3037	519 [M+H-18]^+^ 501 [M+H-18-18]^+^ 391 [M+H-146]^+^ 373 [M+H-146-18]^+^ 355 [M+H-146-18-18]^+^	Frugoside ^b^ 546-02-1	Latex, flowers, leaves, branches, roots	[36,45,46]
**10**	C_29_H_40_O_10_	549.2622	531 [M+H-18]^+^ 513 [M+H-18-18]^+^ 485 [M+H-18-18-28]^+^ 467 [M+H-18-18-28-18]^+^ 405 [M+H-144]^+^ 387 [M+H-144-18]^+^	Calactinic acid ^b^ 24321-45-7	Latex, flowers, leaves, branches, roots	[47]
**11**	C_23_H_34_O_5_	391.2464	373 [M+H-18]^+^ 355 [M+H-18-18]^+^ 337 [M+H-18-18-18]^+^	Coroglaucigenin ^b^ 468-19-9	Latex, flowers, leaves, branches, roots	[36,37]
**12**	C_29_H_44_O_9_	537.3037	519 [M+H-18]^+^ 501 [M+H-18-18]^+^ 375 [M+H-162]^+^ 357 [M+H-162-18]^+^ 339 [M+H-162-18-18]^+^	Desglucouzarin ^b^ 6877-82-3P	Latex, flowers, leaves, branches	[45]
**13**	C_29_H_42_O_9_	535.2901	517 [M+H-18]^+^ 499 [M+H-18-18]^+^ 389 [M+H-146]^+^ 371 [M+H-146-18]^+^ 353 [M+H-146-18-18]^+^	Afroside ^b^ 29010-26-2P	Latex, flowers, leaves, branches, roots	[40]
**14**	C_31_H_41_NO_9_S	604.2544	586 [M+H-18]^+^ 568 [M+H-18-18]^+^ 403 [M+H-201]^+^ 385 [M+H-201-18]^+^	15-hydroxy uscharin ^b^ 29010-26-2	Latex, flowers, leaves, branches, roots	[40]
**15**	C_35_H_54_O_13_	683.3630	521[M+H-162]^+^ 375 [M+H-162-146]^+^ 357 [M+H-162-146-18]^+^ 339 [M+H-162-146-18-18]^+^	*Calotropisprocera*saponin I ^b^ tentatively identified	Latex, flowers, leaves, branches, roots	[41]
**16**	C_29_H_40_O_9_	533.2745	515 [M+H-18]^+^ 497 [M+H-18-18-28]^+^ 387 [M+H-146]^+^ 369 [M+H-146-18]^+^ 351 [M+H-146-18-18]^+^ 323 [M+H-146-18-18-28]^+^	Calactin ^a^ 20304-47-6	Latex, flowers, leaves, branches, roots	[34,35,38,39,42,43,44,48]
**17**	C_29_H_40_O_10_	563.2837	545 [M+H-18]^+^ 513 [M+H-32]^+^ 387 [M+H-176]^+^ 369 [M+H-176-18]^+^ 351 [M+H-176-18-18]^+^ 323 [M+H-176-18-18- 28]^+^	Calactinic acid methylester ^b^ 24211-77-6	Latex, flowers, leaves, branches, roots	[38]
**18**	C_29_H_40_O_9_	533.2745	515 [M+H-18]^+^ 497 [M+H-18-18]^+^ 497 [M+H-18-18-28]^+^ 387 [M+H-146]^+^ 369 [M+H-146-18]^+^ 351 [M+H-146-18-18]^+^ 323 [M+H-146-18-18-28]^+^	Calotropin ^a^ 1986-70-5P	Latex, flowers, leaves, branches, roots	[34,35,36,39,42,43,44,46,48]
**19**	C_31_H_43_NO_8_S	590.2759	572 [M+H-18]^+^ 554 [M+H-18-18]^+^ 526 [M+H-18-18-28]^+^ 387 [M+H-203]^+^ 369 [M+H-203-18]^+^ 351 [M+H-201-18-18]^+^ 323 [M+H-201-18-18-28]^+^	Voruscharin ^b^ 27892-03-1	Latex	[34,35,39,42,43,44,48]
**20**	C_23_H_34_O_4_	375.2519	357 [M+H-18]^+^ 339 [M+H-18-18]^+^ 321 [M+H-18-18-18]^+^	Uzarigenin ^b^ 466-09-1P	Latex, flowers, leaves, branches, roots	[34,36,41,42,49]
**21**	C_31_H_41_NO_9_S	604.2544	586 [M+H-18]^+^ 568 [M+H-18-18]^+^ 540 [M+H-28-18-18]^+^ 387 [M+H-217]^+^ 369 [M+H-217-18]^+^ 351 [M+H-217-18-18]^+^ 323 [M+H-217-18-18-28]^+^	2″-Oxovoruscharin ^b^ 676541-57-4	Latex, roots	[42,48]
**22**		636.2454	618 [M+H-18]^+^ 387 [M+H-247]^+^ 369 [M+ H-247-18]^+^ 351 [M+ H-247-18-18]^+^ 323 [M+ H-247-18-18-28]^+^	Calotropagenin glycoside I ^b^ tentatively identified	Latex, branches, roots	[38]
**23**	C_31_H_42_O_10_	575.2828	557 [M+H-18]^+^ 539 [M+H-18-18]^+^ 497 [M+H-42-18-18]^+^	Asclepin ^b^ 36573-63-4	Latex, flowers, leaves, branches, roots	[36,38]
**24**	C_29_H_38_O_9_	531.2589	513 [M+H-18]^+^ 485 [M+H-18-18]^+^ 467 [M+H-18-18-18]^+^ 387 [M+H-144]^+^ 369 [M+H-144-18]^+^ 351 [M+H-144-18-18]^+^ 323 [M+H-144-18-18-28]^+^	Uscharidin ^b^ 24211-81-2P	Latex, flowers, leaves, branches, roots	[34,36,38,39,43,44]
**25**	C_31_H_43_NO_8_S	590.2759	572 [M+H-18]^+^ 554 [M+H-18-18]^+^ 526 [M+H-18-18-28]^+^ 389 [M+H-201]^+^ 371 [M+ H-201-18]^+^ 353 [M+ H-201-18-18]^+^ 325 [M+ H-201-18-18-28]^+^	Labriformine ^b^ 66419-07-6	Latex	[39]
**26**		634.2667	618 [M+H-18]^+^ 556 [M+H-18-18]^+^ 538 [M+H-18-18-28]^+^ 387 [M+H-215]^+^ 369 [M+ H-215-18]^+^ 351 [M+ H-215-18-18]^+^ 323 [M+ H-215-18-18-28]^+^	Calotropagenin glycoside III ^b^ tentatively identified	Latex, flowers, leaves, branches, roots	[38]
**27**	C_31_H_41_NO_8_S	588.2597	570 [M+H-18]^+^ 552 [M+H-18-18]^+^ 524 [M+H-28-18-18]^+^ 387 [M+H-201]^+^ 369 [M+H-201-18]^+^ 351 [M+H-201-18-18]^+^ 323 [M+H-201-18-18-28]^+^	Uscharin ^a^ 24211-81-2	Latex, leaves	[34,35,36,39,40,42,43,44,48]
**28**		648.2819	387 [M+H-261]^+^ 369 [M+ H-261-18]^+^ 351 [M+ H-261-18-18]^+^ 323 [M+ H-261-18-18-28]^+^	Calotropagenin glycoside IV ^b^ tentatively identified	Latex	[38]
**29**		602.2391	584 [M+H-18]^+^ 566 [M+H-80]^+^ 387 [M+H-249]^+^ 369 [M+ H-249-18]^+^ 351 [M+ H-249-18-18]^+^ 323 [M+ H-249-18-18-28]^+^	Calotropagenin glycoside II ^b^ tentatively identified	Latex	[38]
**Flavonoids**						
**30**	C_27_H_30_O_16_	611.1607	465 [M+H-146]^+^ 303 [M+H-146-162]^+^	Rutin ^a^ 153-18-4	Flowers, leaves, branches, roots	[50]
**31**	C_27_H_30_O_16_	611.1607	479 [M+H-132]^+^ 317 [M+H-132-162]^+^	Isorhamnetin-hexoside-pentoside ^b^ tentatively identified	Flowers, leaves	MS data
**32**	C_27_H_30_O_15_	595.1657	449 [M+H-146]^+^ 287 [M+H-146-162]^+^	Kaempferol-robinoside ^a^ 17297-56-2	Flowers, leaves, branches, roots	standard
**33**	C_28_H_32_O_16_	625.1763	479 [M+H-146]^+^ 317 [M+H-146-162]^+^	Isorhamnetin-robinoside ^b^ 107740-46-5	Flowers, leaves, branches, roots	[42]
**34**	C_27_H_30_O_15_	595.16575	449 [M+H-146]^+^ 287 [M+H-146-162]^+^	Kaempferol-rutinoside ^a^ 17650-84-9	Flowers, leaves, branches, roots	standard
**35**	C_28_H_32_O_16_	625.1763	479 [M+H-146]^+^ 317 [M+H-146-162]^+^	Isorhamnetin-rutinoside ^b^ 604-80-8	Flowers, leaves, branches, roots	[42]
**36**	C_22_H_22_O_12_	479.1171	317 [M+H-162]^+^	Isorhamnetin-Hexoside ^b^ 1456622-02-8	Flowers, leaves, branches, roots	MS data
**37**	C_21_H_20_O_11_	449.1066	287 [M+H-162]^+^	Kaempferol-Hexoside ^a^ 1108717-10-7	Flowers, leaves, roots	MS data

^a^: compound identification compared with a standard, ^b^: compound identification according to MS and literature data.

**Table 2:** Technical and validation parameters of the LC/MS-based method used for the quantization of calactin, 15-hydroxy calactin, rutin, and isorhamnetin glycoside.

Compound	MRM Transition	LloQ (ng/mL)	Accuracy (%)	Precision (%)	Linearity (0–24 µg/mL)
Calactin	533/323	25	91	88	0.9987
15-Hydroxy calactin	549/199	25	85	81	0.9991
Rutin	611/303	10	92	85	0.9976
Isorhamnetin glycoside	625/317	100	90	87	0.9977

## Data Availability

The data presented in this study are available on request from the corresponding author.

## Supplementary Materials

The following are available online, Figure S1: 1H NMR spectrum of MeOH Extract of leaves, Figure S2. 1H NMR spectrum of MeOH Extract of flowers; Figure S3. 1H NMR spectrum of MeOH Extract of stems; Figure S4. 1H NMR spectrum of MeOH Extract of roots; Figure S5. 1H NMR spectrum of Latex Extract.

## Sample Availability

Samples of the calactin, rutin, 15-β-OH-calactin, isorhamnetin glycoside are available from the authors.
