# Supplementary material for: Systematic Phytochemical Screening of Different Organs of Calotropis procera and the Ovicidal Effect of Their Extracts to the Foodstuff Pest Cadra cautella
Source: Molecules. 2021 Feb 9;26(4):905. doi: 10.3390/molecules26040905 (PMC7916075; doi:10.3390/molecules26040905)
Supplement: Supplementary file 1 [file molecules-26-00905-s001.pdf]

Supporting Information

Systematic phytochemical screening of different organs of *Calotropis procera* and the ovicidal effect of their extracts to the foodstuff pest *Cadra cautella*.

Ammar Bader[1], Ziad Omran[1,5], Ahmed I. Al-Asmari[2, *], Valentina Santoro[3], Nunziatina De Tommasi[3], Massimiliano D'Ambola[3], Fabrizio Dal Piaz[3], Barbara Conti[4, *], Stefano Bedini[4] and Majed Halwani[5, *]

1 Department of Pharmacognosy, Faculty of Pharmacy, Umm Al-Qura University, Makkah, 21955, Saudi Arabia; ambader@uqu.edu.sa; zhomran@uqu.edu.sa
2 King Abdulaziz Hospital, Laboratory Department, Jeddah, Saudi Arabia; ahmadalasmari@yahoo.com
3 Dipartimento di Farmacia, Università di Salerno, Fisciano (SA), Italy; vsantoro@unisa.it; detommasi@unisa.it; mdambola@unisa.it; fdalpiaz@unisa.it
4 Department of Agriculture, Food and Environment, University of Pisa, Via del Borghetto 80, Pisa, Italy; barbara.conti@unipi.it; stefano.bedini@unipi.it
5 Nanomedicine Department, King Abdullah International Medical Research Center, King Saud Bin Abdulaziz University for Health Sciences, Riyadh, Saudi Arabia; halawanima@ngha.med.sa

* Correspondence: ahmadalasmari@yahoo.com, Tel.: +9665599155725 (A.I.A.); barbara.conti@unipi.it, Tel.: +390502216125 (B.C.) and halawanima@ngha.med.sa, Tel.: +966114294433 (MH).

Content:

Page No.

2 Extraction and Isolation method

3 Figure S1. ^{1}H NMR spectrum of MeOH Extract of leaves

4 Figure S2. ^{1}H NMR spectrum of MeOH Extract of flowers

5 Figure S3. ^{1}H NMR spectrum of MeOH Extract of stems

6 Figure S4. ^{1}H NMR spectrum of MeOH Extract of roots

7 Figure S5. ^{1}H NMR spectrum of Latex Extract

Extraction and Isolation*:* Dried and powdered leaves of *C. procera* (500 g) were extracted with MeOH-H_2O to give 33.0 g of crude methanolic extract. The *C. procera* obtained crude extract was dissolved in water and successively partitioned with *n*-hexane, and *n*-butanol, to yield 6.0 g and 11.5 g, respectively. The *n*-butanol fraction was subjected to flash silica gel CC using on Isolera Biotage (SNAP 340 g column, flow rate 90 mL/min), eluting with $CHCl_3$ followed by increasing concentrations of MeOH in $CHCl_3$ (between 1 and 100%) collecting 27 mL of fractions, that were grouped by TLC into 10 major fractions (A-J). Fractions C (95.6.0 mg) and D (205.6 mg) were subjected to RP-HPLC with MeOH-H_2O (5:5) to yield calotropin (1.1 mg, t_R 31 min) and calactin (3.8 mg, t_R 18 min) from fraction C, and uscharin (0.9 mg, t_R 31.5 min) from fraction D.

Fraction F (239.7 mg) was purified by RP-HPLC with MeOH-H_2O (1:1) to afford compound 16β-hydroxy-calactin (1.2 mg, t_R 15 min) and isorhamnetin **(**1.2 mg, t_R 26 min).

Fractions G (56.0 mg), and H (181.0 mg), were separately subjected to RP-HPLC with MeOH-H_2O (3:2) to yield 15β-hydroxy-calactin (2.8 mg, t_R 15 min), and 12β-hydroxy-calactin (1.2 mg, t_R 18 min) from fraction G, 15β-hydroxy-calactin (1.7 mg, t_R 15 min) and calactin (2.5 mg, t_R 25 min) from fraction H.

Fractions I (93.0 mg) and J (201.0 mg) were individually chromatographed by RP-HPLC with MeOH-H_2O (3.5:6.5) to give rutin (2.9 mg, t_R 12 min) and kampferol (1.0 mg, t_R 15 min) from fraction I and rutin (4.2 mg, t_R 12 min), and isorhamnetin (3.7 mg, t_R 21 min) from fraction J.

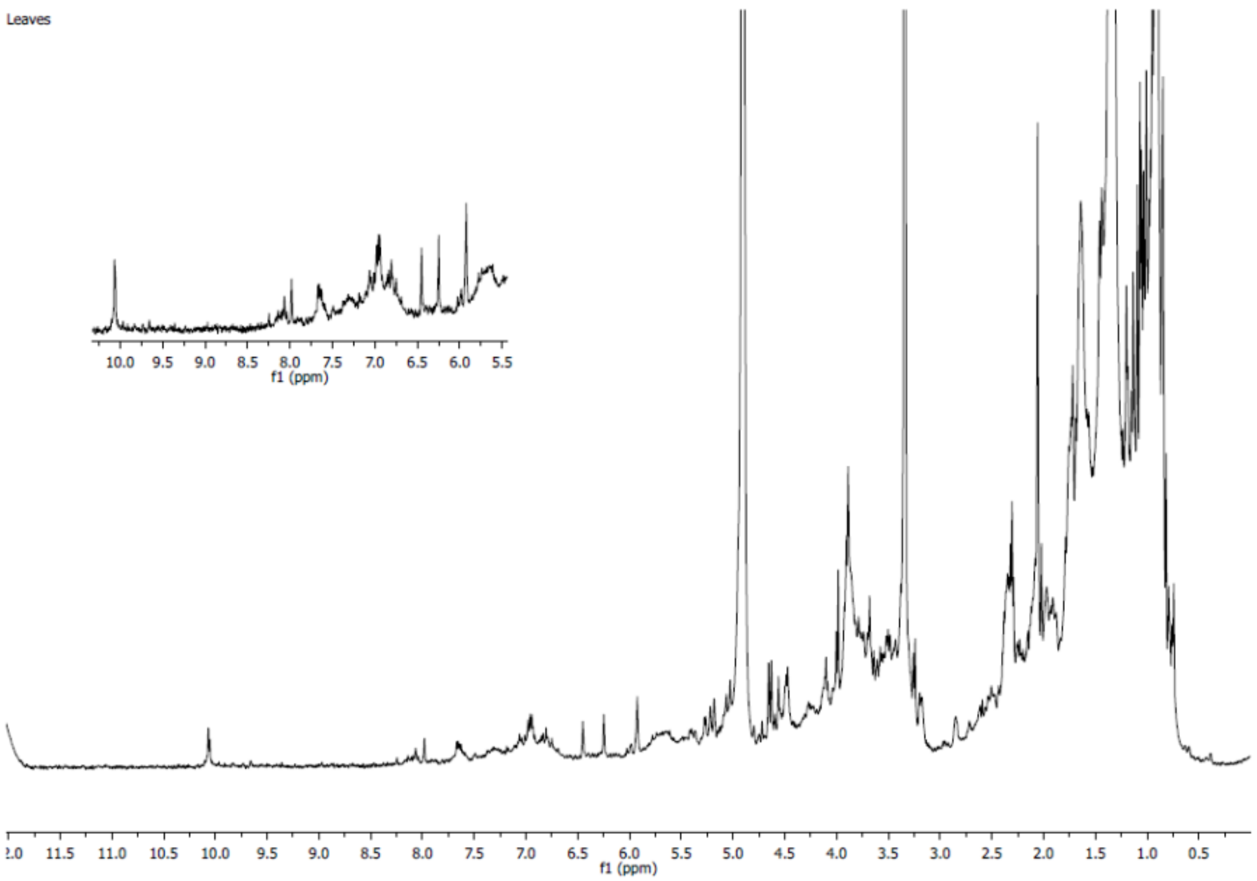

Figure S1. ^{1}H NMR spectrum of MeOH Extract of leaves (CD_3OD, 500 MHz)

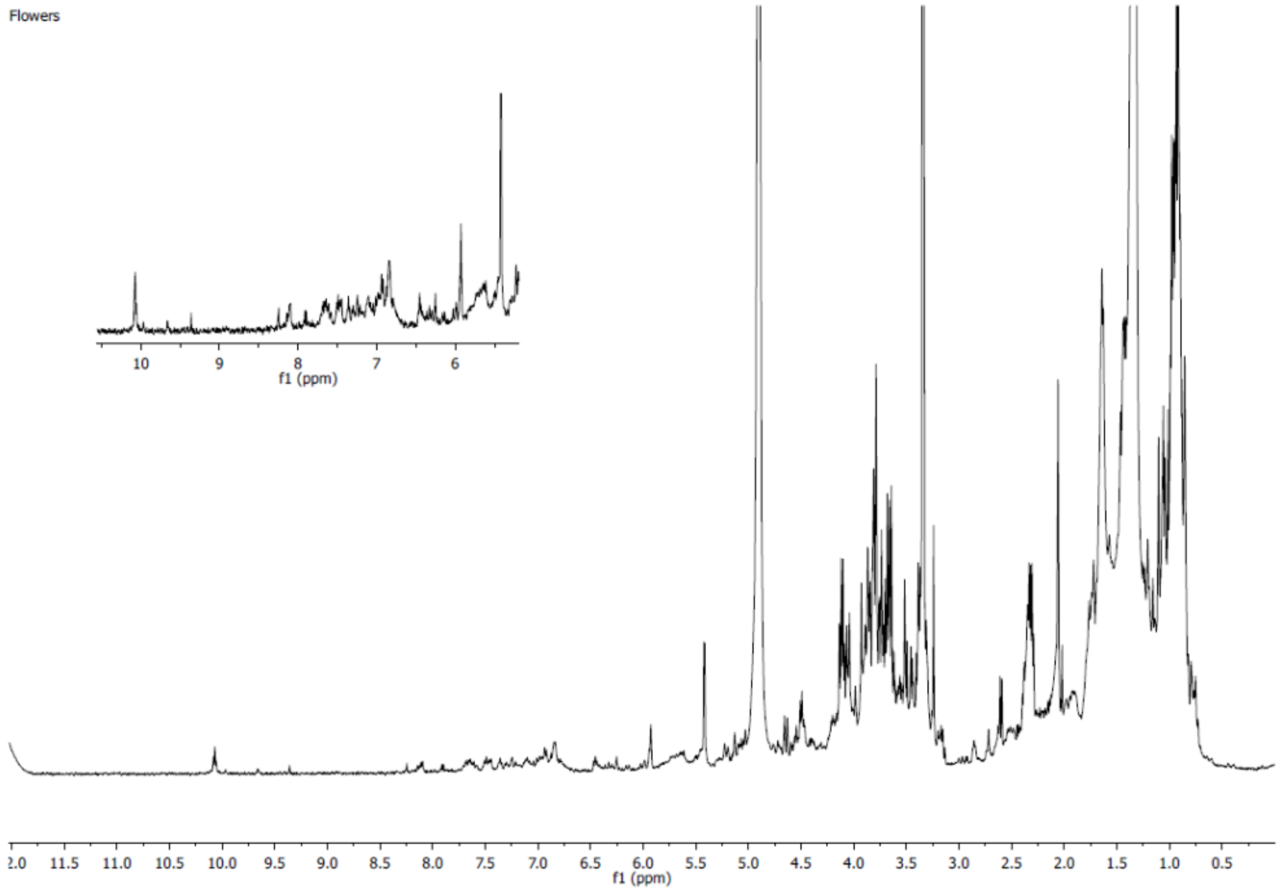

Figure S2. ^{1}H NMR spectrum of MeOH Extract of flowers (CD_3OD, 500 MHz)

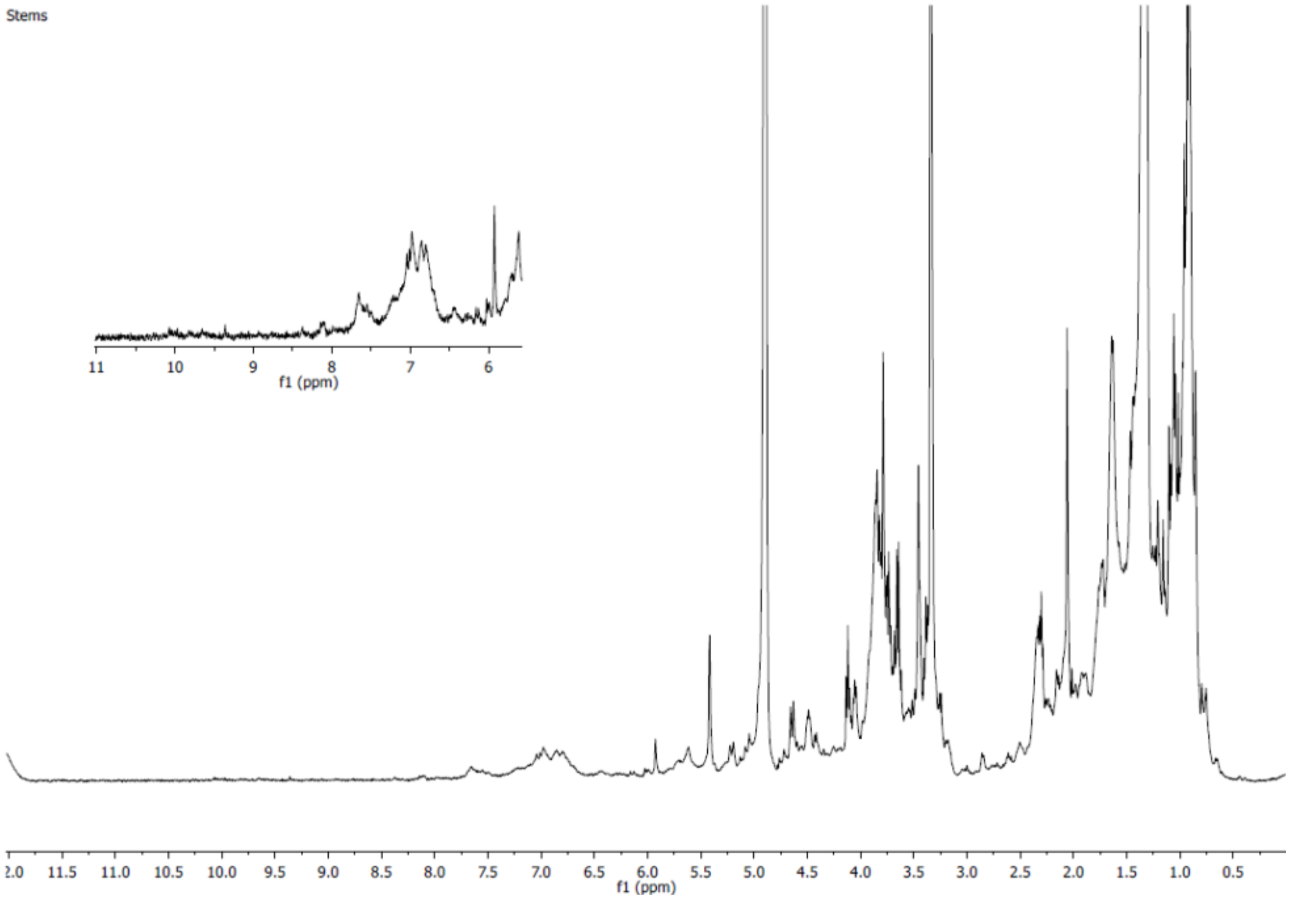

Figure S3. ^{1}H NMR spectrum of MeOH Extract of stems (CD_3OD, 500 MHz)

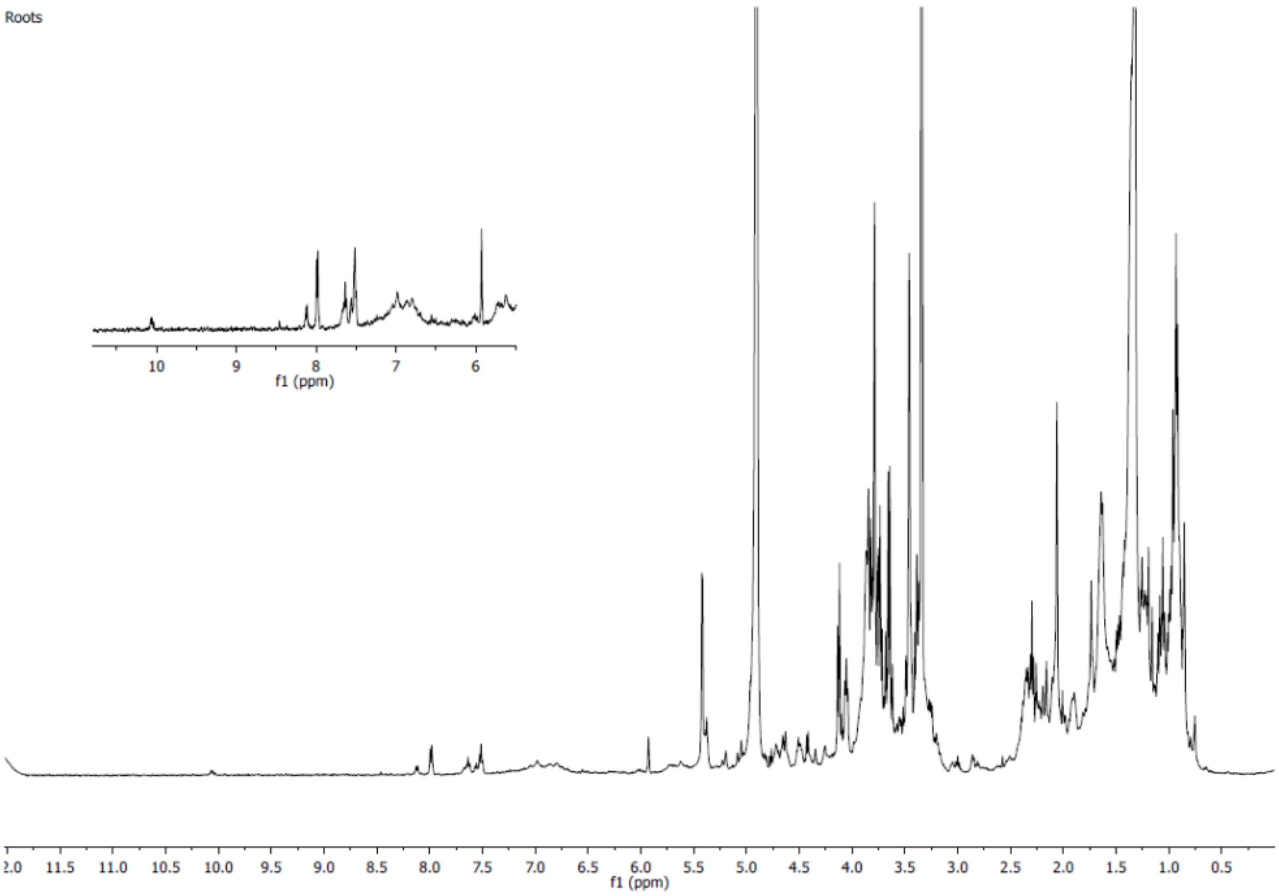

Figure S4. ^{1}H NMR spectrum of MeOH Extract of roots (CD_3OD, 500 MHz

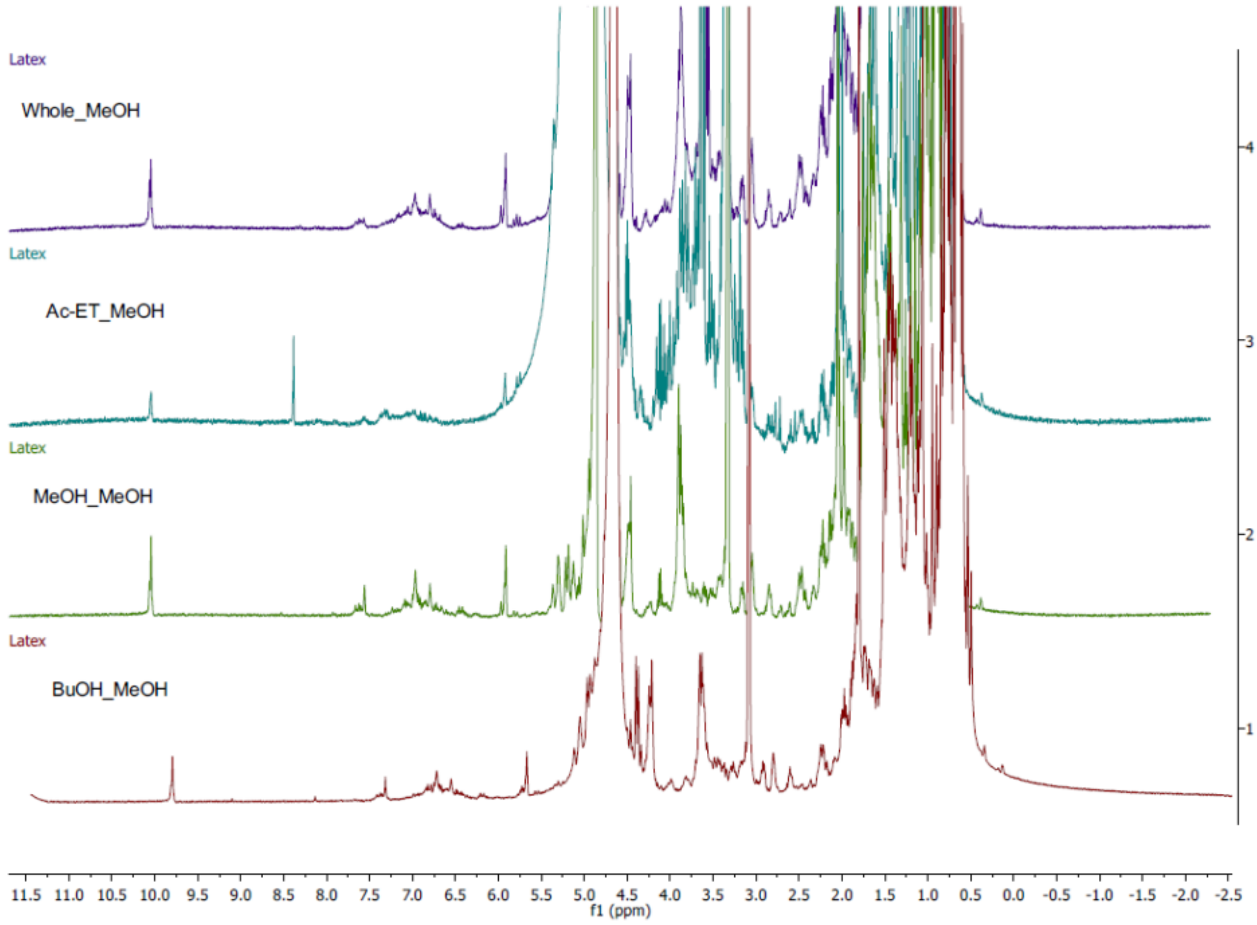

Figure S5. ^{1}H NMR spectrum of Latex Extract (CD_3OD, 500 MHz
